# Untersuchung standardisierter Anamnesefragebögen zur Diagnostik und Differenzierung von obstruktiven und klaffenden Tubenfunktionsstörungen

**DOI:** 10.1007/s00106-020-00931-z

**Published:** 2020-09-03

**Authors:** J. Lönnecker, N. M. Weiss, A. Heinrichs, R. Mlynski, S. Rettschlag

**Affiliations:** Klinik für Hals-Nasen-Ohrenheilkunde, Kopf- und Halschirurgie „Otto Körner“, Universitätsmedizin Rostock, Doberaner Straße 137–139, 18057 Rostock, Deutschland

**Keywords:** Funktionsstörung der Eustachischen Röhre, Differentialdiagnose, Verlaufskontrolle, ETDQ‑7, PHI-10, Eustachian tube dysfunction, Differential diagnosis, Follow-up, ETDQ‑7, PHI-10

## Abstract

**Hintergrund:**

Eine klaffende Tube kann insbesondere durch Autophonie, Druckgefühl und gestörten Höreindruck zu einer Einschränkung der Lebensqualität führen. Bei fehlenden spezifischen Symptomen kann die Diagnose der klaffenden Tube schwierig sein. Insbesondere die Abgrenzung zur chronisch obstruktiven Tubenfunktionsstörung stellt eine Herausforderung dar. Da derzeit kaum standardisierte Diagnostik- und Therapieoptionen zur Verfügung stehen, ist eine strukturierte Untersuchung zur sicheren Diagnostik und wissenschaftlichen Aufarbeitung dieser Erkrankung erforderlich. Für die Diagnostik der chronisch obstruktiven Tubenfunktionsstörung wurde 2012 bereits der „Eustachian Tube Dysfunction Questionnaire“ (ETDQ-7-Fragebogen) nach McCoul entwickelt. Für die klaffende Tube existiert seit 2017 der PHI-10-Fragebogen („patulous Eustachian tube handicap inventory“) nach Kobayashi.

**Material und Methoden:**

Der PHI-10-Fragebogen wurde ins Deutsche übersetzt und an 41 Gesunden, 13 Patienten mit Tinnitus auris, 11 Patienten mit klaffender Tube und 18 Patienten mit chronisch obstruktiver Tubenventilationsstörung getestet. Zusätzlich erfolgte im Vergleich die Auswertung des ETDQ‑7 nach McCoul.

**Ergebnisse:**

Es erfolgt die Präsentation der deutschen Übersetzung des PHI-10 und der Ergebnisse von PHI-10 und ETDQ‑7 in allen Patientengruppen. Der ETDQ‑7 hat das Risiko falsch-positiver Ergebnisse bei Patienten mit klaffender Tube und der PHI-10 bei Patienten mit obstruktiver Tubenfunktionsstörung. Beide untersuchten Fragebögen sind falsch-positiv bei Tinnituspatienten.

**Schlussfolgerung:**

Der PHI-10 (deutsch) und ETDQ‑7 (deutsch) sind eine nützliche Unterstützung der Anamnese bezüglich Tubenfunktionsstörungen. Sie unterscheiden jedoch nur unzureichend zwischen klaffenden und obstruktiven Tubenfunktionsstörungen und eignen sich nicht für Patienten mit Tinnitus. Die Stärke der Fragebögen ist in der Verlaufskontrolle und dem Monitoring von Therapieergebnissen zu sehen.

## Abstract (en)

**Background:**

Patulous Eustachian tube (ET) dysfunction can impair quality of life (QOL) due to autophony, pressure sensation, and an altered impression of sound. In cases of nonspecific complaints the diagnosis of patulous ET can be difficult, and its distinction from chronic obstructive ET dysfunction is particularly challenging. Since there is currently a lack of standardized diagnostic and therapeutic options, a structured diagnostic workup is essential for accurate diagnosis of this condition. The Eustachian Tube Dysfunction Patient Questionnaire (ETDQ-7) was established by McCoul et al. in 2012 for investigating chronic obstructive ET dysfunction. The Patulous Eustachian Tube Handicap Inventory-10 (PHI-10) was published by Kobayashi et al. in 2017 as an instrument to evaluate patulous ET.

**Patients and methods:**

The PHI-10 questionnaire was translated into German and validated in a total of 83 patients (41 healthy subjects, 13 patients with tinnitus, 11 patients with patulous ET, and 18 patients with chronic obstructive ET dysfunction). In addition, the PHI-10 (German) was compared to ETDQ‑7.

**Results:**

The results of the English version of the PHI-10 questionnaire could be confirmed using the German version. The German version of the PHI-10 and the results of the PHI-10 and ETDQ‑7 for each group of patients are presented. The ETDQ‑7 leads to false-positive results in patients with patulous ET and the PHI-10 generates false-positive results in patients with chronic obstructive ET dysfunction. Both questionnaires result in false-positive results in patients with tinnitus.

**Conclusion:**

The PHI-10 and ETDQ‑7 (German) can be recommended as an additional tool for preoperative assessment of ET dysfunction. However, they insufficiently discriminate between patulous and obstructive ET dysfunction and are not suitable for patients with tinnitus. The strength of the two questionnaires lies in their suitability for use in monitoring therapeutic success during follow-up.

## Hintergrund

In den letzten Jahren ist eine Zunahme von Patienten mit Verdacht auf eine Funktionsstörung der Tuba auditiva zu bemerken. Diese Patienten beklagen häufig eher diffuse Beschwerden. Vorrangige Symptome sind ein gestörter Höreindruck, Autophonie, Druckgefühl auf den Ohren, Ohrgeräusche und Probleme beim Druckausgleich. Grundsätzlich sind die Differenzialdiagnosen im Bereich der Tuba auditiva eine chronisch obstruktive Tubenfunktionsstörung, eine klaffende Tube oder eine situative Tubenfunktionsstörung[20]. Wichtigste Differenzialdiagnosen sind der Tinnitus auris, die Schallempfindungsschwerhörigkeit und die Dehiszenz des oberen Bogengangs.

Die Anamnese ist der erste wichtige Schritt auf dem Weg zur Diagnose und Therapieempfehlung. Der Patient sollte dabei nach Situationen gefragt werden, in denen die Beschwerden zunehmen oder sich verbessern. Der Stellenwert der Anamnese ist auch deshalb so hoch, weil bisher kein diagnostischer Goldstandard für Funktionsstörungen der Tuba auditiva vorliegt. Zur Diagnostik zu empfehlen sind die Ohrmikroskopie mit dem Valsalva-Manöver, die pneumatische Otoskopie und der Toynbee-Versuch. Der Patient sollte unter ohrmikroskopischer Kontrolle zur forcierten nasalen Atmung bei komprimiertem kontralateralem Nasenloch aufgefordert werden, um eine atemsynchrone Trommelfellbewegung aufzudecken. Eine Objektivierung der Funktion der Eustachischen Röhre mittels apparativer Untersuchungen gestaltet sich weiterhin schwierig[10]. Verfahren wie die Sonotubometrie[11], Tubenmanometrie nach Estève[1] mit dem Tubenscore[21] oder Langzeitmessungen mit Dehnungsstreifen auf dem Trommelfell [5] werden aktuell hinsichtlich ihrer Zuverlässigkeit untersucht. Derzeit gibt es keine grundsätzlichen in Leitlinien und Übersichtsarbeiten empfohlenen Diagnostikverfahren und keine klare Empfehlung zur objektiven Beurteilung der Funktion der Eustachischen Röhre [8, 13, 20].

In den letzten Jahren wurden diverse neue Verfahren zur Therapie von Tubenfunktionsstörungen entwickelt. Zur Behandlung der obstruktiven Tubenfunktionsstörung stehen z. B. die Ballontuboplastie [16, 24] sowie die Lasertuboplastie [7] zur Verfügung. Zur Behandlung der klaffenden Tube werden z. B. die Patulous Eustachian Tube Reconstruction [15], der Kobayashi Plug [19] oder die Unterspritzung des Torus tubaris mit Vox®-Implants (Uroplasty BV, DC Geleen, Niederlande) [23] vorgeschlagen. Unabhängig von der Art der Tubenfunktionsstörung und der geplanten Therapie ist eine sichere und präzise Funktionsdiagnostik der Eustachischen Röhre für ein sinnvolles therapeutisches Vorgehen essenziell. Um dieser Problemsituation zu begegnen, gab es Bemühungen, die Anamnese bei Tubenfunktionsstörungen zu strukturieren, und es wurden anamnesebasierte Scoringsysteme für die obstruktive und klaffende Tubenfunktionsstörung entwickelt.

Beim Eustachian Tube Dysfunction Questionnaire (ETDQ-7) handelt es sich um einen 2012 entwickelten Fragebogen zur Untersuchung von Patienten mit obstruktiver Tubenfunktionsstörung. Er besteht aus 7 Fragen mit je einer numerischen Antwortskala von 1 (kein Problem) bis 7 (schwerstes Problem). Ein Wert ab ≥14,5 Punkten gilt als pathologisch [12]. Beim „patulous Eustachian tube handicap inventory“ (PHI-10) handelt es sich um einen Fragebogen zur Erfassung der Symptome bei klaffender Tube. Der PHI-10 wurde in Anlehnung am das Tinnitus Handicap Inventory (THI-12) erstellt. Er enthält 10 Fragen bezogen auf die letzten 4 Wochen, und die Einteilung des Schweregrads der klaffenden Tube richtet sich nach dem erreichten Gesamtpunktwert. Jede einzelne Frage kann mit „ja“, entsprechend 4 Punkten, „manchmal“, entsprechend 2 Punkten, und „nein“, entsprechend 0 Punkten, beantwortet werden. Ein Gesamtpunktwert von 0–8 wird als keine Beeinträchtigung, ein Gesamtpunktwert von 10–16 als milde Beeinträchtigung, ein Gesamtpunktwert von 18–24 als mäßige Beeinträchtigung und ein Gesamtpunktwert von 26–40 als schwere Beeinträchtigung gewertet [4].

Ziel dieser Studie war es, den neu entwickelten erkrankungsspezifischen Fragebogen zur Erfassung von Symptomen einer klaffenden Tube (PHI-10) in deutscher Übersetzung zu validieren. Die Ergebnisse des PHI-10 und des erkrankungsspezifischen Fragebogens für die obstruktive Tubenfunktionsstörung (ETDQ‑7) sollen an einem Kollektiv mit klaffender Tube, obstruktiver Tubenfunktionsstörung, Tinnitus auris und Gesunden ausgewertet werden.

## Patienten und Methoden

Wir verwendeten den ETDQ‑7 (Tab. 1) in der deutschen Übersetzung und übersetzten zusätzlich den PHI-10-Fragebogen ins Deutsche (Tab. 2). Anschließend wendeten wir die beiden Fragebögen bei 41 Gesunden (80 Ohren) und 18 Patienten (32 Ohren) mit Nachweis einer chronisch obstruktiven Tubenfunktionsstörung, 13 Patienten (22 Ohren) mit Tinnitus und Ausschluss einer Tubenfunktionsstörung sowie 11 Patienten (11 Ohren) mit klaffender Tube an. Die Patienten erhielten eine Ohrmikroskopie mit Valsalva-Versuch, eine Epipharyngoskopie, eine Tubenmanometrie mit Bestimmung des Eustachian Tube Score (ETS‑7; Tab. 3) und ein Tympanogramm. Als gesunde Kontrollgruppe verwendeten wir freiwillige Probanden mit unauffälligem Ohrbefund und unauffälliger Ohranamnese, freiem Epipharynx und einem ETS-7 > 7. Diese wurden als tubengesund eingestuft. In der Gruppe der Patienten mit chronisch obstruktiver Tubenfunktionsstörung waren Patienten mit Tympanogramm Typ B/C und/oder rezidivierenden Paukenergüssen in den letzten 2 Jahren und/oder subjektiv und objektiv nicht durchführbarem Valsalva-Manöver sowie einem ETS-7 ≤ 7. Die Patienten mit klaffender Tube wurden durch eine typische Anamnese und eine atemsynchrone Trommelfellbewegung unter dem Ohrmikroskop identifiziert. Bei Patienten mit Tinnitus auris wurde ein unauffälliger Ohrbefund, ein freier Epipharynx und ein ETS-7 > 7 als Ausschluss einer Tubenfunktionsstörung angesehen.

**Table 1:** 

Beschwerden in den letzten 4 Wochen an den meisten Tagen	Kein Problem		Mäßiges Problem			Schweres Problem	
1. Druckgefühl im Ohr	1	2	3	4	5	6	7
2. Schmerzen in den Ohren	1	2	3	4	5	6	7
3. Gefühl, die Ohren seien verstopft oder „unter Wasser“	1	2	3	4	5	6	7
4. Ohrenbeschwerden im Rahmen von Erkältungen und Nebenhöhlenentzündungen	1	2	3	4	5	6	7
5. Knisternde oder knallende Geräusche in den Ohren	1	2	3	4	5	6	7
6. Klingeln in den Ohren	1	2	3	4	5	6	7
7. Gefühl, das Hören sei dumpf	1	2	3	4	5	6	7

**Table 2:** 

Beschwerden in den letzten 4 Wochen	Ja	Manchmal	Nein
1. Haben Sie aufgrund Ihrer Beschwerden Konzentrationsstörungen?	4	2	0
2. Führen Ihre Symptome dazu, dass Sie andere Menschen schlechter hören können?	4	2	0
3. Machen Ihre Symptome Sie ärgerlich?	4	2	0
4. Fühlen Sie sich, als könnten Sie Ihren Beschwerden nicht entkommen?	4	2	0
5. Stören Ihre Beschwerden Ihre Fähigkeit, soziale Aktivitäten zu genießen?	4	2	0
6. Fühlen Sie sich aufgrund Ihrer Beschwerden frustriert?	4	2	0
7. Stören Ihre Beschwerden Ihre berufliche Tätigkeit oder Verrichtungen im Haushalt?	4	2	0
8. Haben Sie den Eindruck, dass Ihre Beschwerden Stress in Ihre Beziehung zu Familie und Freunden gebracht haben?	4	2	0
9. Finden Sie es schwer, sich auf etwas anderes als Ihre Beschwerden zu konzentrieren?	4	2	0
10. Beängstigen Ihre Symptome Sie?	4	2	0

**Table 3:** 

Symptom/Befund	2 Punkte	1 Punkt	0 Punkte
Knacken im Ohr beim Schlucken (Toynbee)	Ja, immer	Manchmal	Nein, niemals
Positiver Valsalva	Ja, immer	Manchmal	Nein, niemals
TMM 30 mbar	R ≤ 1	R > 1	Kein R
TMM 40 mbar	R ≤ 1	R > 1	Kein R
TMM 50 mbar	R ≤ 1	R > 1	Kein R
Objektiver Valsalva	Deutlich positiv	Schwach/verzögert	Negativ
Tympanogramm (Jerger)	Typ A	Typ C	Typ B

Statistische Analysen wurden mit SPSS® (Version 25.0, IBM, NY, USA) durchgeführt. Zur Berechnung der Signifikanz wurde der Mann-Whitney-U-Test durchgeführt. Vor der Datenerhebung wurde ein Signifikanzniveau von a = 0,05 festgelegt. Bei einem *p*-Wert <0,05 wurde von einem signifikanten Ergebnis ausgegangen. Ein *p*-Wert von <0,01 wurde als sehr signifikant bewertet, und ein Wert <0,001 wurde als hoch signifikant interpretiert. Zur Beurteilung der diagnostischen Güte der Fragebögen wurden jeweils die Sensitivität, die Spezifität sowie die positiven und negativen prädiktiven Werte errechnet. Des Weiteren wurden zur Untersuchung der Gesamtgenauigkeit der Fragebögen ROC-Analysen erstellt und die Fläche unter der Kurve (AUC) berechnet.

## Ergebnisse

In der Gruppe der Gesunden waren 71 % weiblich und 29 % männlich. Das Durchschnittsalter lag bei 24 Jahren (18–35 Jahre). Der Valsalva-Versuch war bei 68 % der Ohren positiv und bei 31 % schwach positiv. Nur eines der Ohren wies ein Typ-B-Tympanogramm nach Jerger auf, alle anderen einen Typ A. Der ETS‑7 lag im Mittel bei 12,24 Punkten und somit im Gesunden. Kein Patient wies einen pathologischen ETS‑7 auf. Im ETDQ‑7 ergab sich ein Mittelwert von 8,71 Punkten und beim PHI-10 von 0,53 Punkten. Damit waren beide Scores im Mittel nicht pathologisch.

In der Gruppe der Patienten mit chronisch obstruktiver Tubenfunktionsstörung wurden 18 Patienten (32 Ohren) untersucht. Das Durchschnittsalter lag bei 40 Jahren (20–65 Jahre). Es waren 72 % der Patienten weiblich und 28 % männlich. Bei 88 % der untersuchten Ohren war der Valsalva-Versuch negativ. Bei 6 % der Ohren ist er positiv ausgefallen, und bei 6 % war er schwach positiv. 41 % der Ohren zeigten ein Tympanogramm Typ B, 25 % ein Typ A und 34 % einen Typ C nach Jerger. Der ETS-7-Score ergab einen mittleren Gesamtpunktwert von 4,59. Im ETDQ‑7 ergab sich ein Mittelwert von 24,06 Punkten und beim PHI-10 von 19,31 Punkten. Damit waren beide im Mittel pathologisch. Bei 75 % der Ohren wies der ETDQ‑7 Werte für eine chronisch obstruktive Tubenfunktionsstörung auf. Bei 56 % der Ohren von Patienten mit diagnostizierter obstruktiver Tubenfunktionsstörung bestand laut PHI-10 Anhalt für mindestens eine mäßig klaffende Tube (18–40 Punkte).

Insgesamt 11 Patienten (11 Ohren) wurden in der Gruppe der klaffenden Tube untersucht. Im Durchschnitt waren die Patienten 50 Jahre alt (32–72 Jahre). Es waren 55 % der Patienten weiblich und 45 % männlich. Der ETDQ‑7 ergab einen mittleren Gesamtpunktwert von 23,0 und der PHI-10 von 28,18. Damit ergab sich bei beiden Scores im Mittel ein pathologisches Ergebnis. Bei 91 % der Ohren wäre laut ETDQ‑7 eine chronisch obstruktive Tubenfunktionsstörung zu diagnostizieren gewesen. Bei allen untersuchten Ohren bestand laut PHI-10 Anhalt für eine mindestens mäßig schwer ausgeprägte klaffende Tube.

In der Gruppe der Patienten mit Tinnitus gingen 13 Patienten (22 Ohren) in die Untersuchung ein. Das Durchschnittsalter lag bei 55 Jahren (22–73 Jahre). Es waren 54 % der Patienten weiblich und 46 % männlich. Der Valsalva-Versuch war bei 72 % der untersuchten Ohren positiv, bei 23 % schwach positiv und bei 5 % negativ. Bei 86 % der Ohren zeigte sich ein Tympanogramm Typ A, bei 5 % ein Typ B und bei 9 % ein Typ C. Der ETS‑7 lag mit durchschnittlich 11,09 Punkten im Gesunden. Im ETDQ‑7 errechnete sich ein Mittelwert von 17,91 Punkten und beim PHI-10 von 20,82 Punkten. Somit ergab sich im Mittel bei beiden Fragebögen ein pathologisches Ergebnis. Bei 59,09 % der Ohren wäre laut ETDQ‑7 eine chronisch obstruktive Tubenfunktionsstörung zu diagnostizieren gewesen, und bei 72,73 % bestand laut PHI-10 Anhalt für eine mindestens mäßig ausgeprägte klaffende Tube.

**Figure 1:**
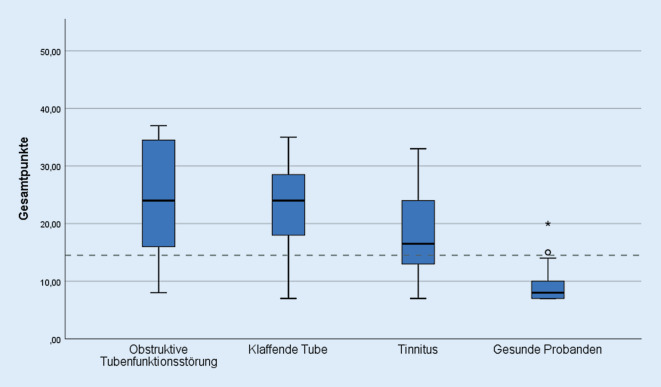

**Figure 2:**
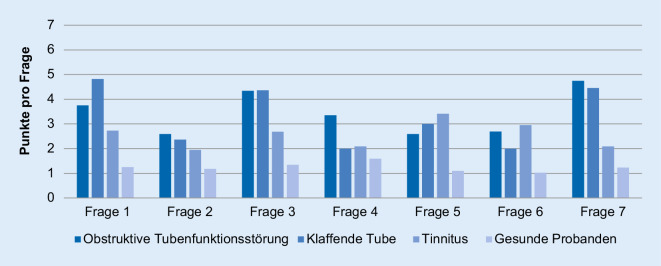

Die Auswertung der Ergebnisse des ETDQ‑7 in allen Patientengruppen ist Abb. 1 zu entnehmen. Die Mehrheit der Gesamtpunktwerte der Patienten mit obstruktiver und klaffender Tubenfunktionsstörung sowie der Tinnituspatienten lagen im pathologischen Bereich (≥14,5 Punkte). Der mittlere Gesamtpunktwert des ETDQ‑7 fiel in allen 3 Studiengruppen signifikant höher aus als bei den gesunden Probanden (*p* < 0,001). Die Patienten mit obstruktiver Tubenfunktionsstörung erzielten im Durchschnitt den höchsten Punktwert. Dieses Ergebnis unterscheidet sich nicht signifikant von dem Testergebnis der Patienten mit klaffender Tube (*p* = 0,675). Die Abb. 2 schlüsselt die Ergebnisse des ETDQ‑7 getrennt nach Einzelfragen auf. Es zeigt sich, dass sich die Einzelpunktwerte der Patienten mit obstruktiver und klaffender Tubenfunktionsstörung bis auf Frage 4 (*p* = 0,026) nicht signifikant voneinander unterscheiden. Die Einzelpunktwerte der Patienten mit obstruktiver Tubenfunktionsstörung und die der Tinnituspatienten unterscheiden sich mit Ausnahme der Fragen 3, 4 und 7 ebenfalls nicht signifikant voneinander. In der ROC-Analyse ergibt sich für die Diagnosestellung einer obstruktiven Tubenfunktionsstörung eine Fläche unter der Kurve von 0,959 (95%-Konfidenzintervall von 0,922–0,995). Es errechnet sich in dieser Analyse des ETDQ‑7 zur Diagnosestellung einer obstruktiven Tubenfunktionsstörung eine Sensitivität von 75 % und eine Spezifität von 95 %. Der positive prädiktive Wert beträgt 86 % und der negative prädiktive Wert beträgt 90 %. Für die Unterscheidung von klaffender und obstruktiver Tubenfunktionsstörung errechnet sich eine Fläche unter der Kurve von 0,543 (95%-Konfidenzintervall von 0,36–0,725).

**Figure 3:**
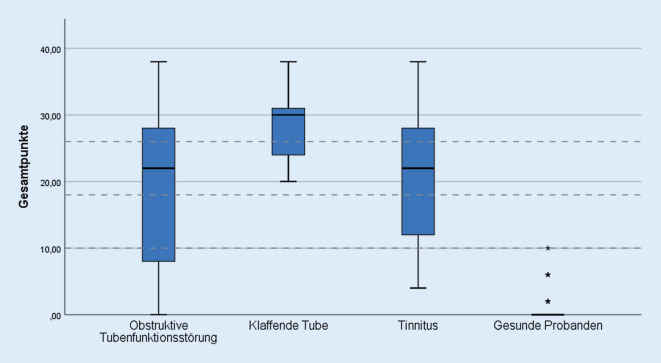

**Figure 4:**
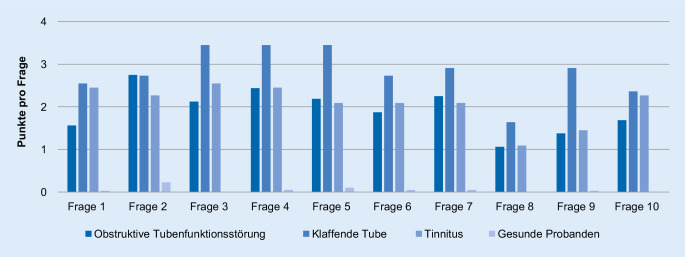

Die Verteilung der Gesamtpunktwerte des PHI-10 wird in Abb. 3 dargestellt. Die Mehrheit der Gesamtpunktwerte der Patienten mit obstruktiver und klaffender Tubenfunktionsstörung sowie der Tinnituspatienten lag im pathologischen Bereich. Die mittleren Gesamtpunkte der Patienten mit klaffender und obstruktiver Tubenfunktionsstörung sowie die der Tinnituspatienten fallen signifikant höher aus als die der gesunden Probanden (*p* < 0,001). Patienten mit klaffender Tube erreichten im Mittel die höchsten Gesamtpunkte und lagen im Bereich einer schwer ausgeprägten klaffenden Tube (26–40 Punkte). Die mittleren Punktwerte der Patienten mit obstruktiver Tubenfunktionsstörung sowie die der Tinnituspatienten liegen ebenfalls im pathologischen Bereich einer mäßig ausgeprägten klaffende Tube (18–24 Punkte). Zur genaueren Auswertung des PHI-10 wurden die Einzelfragen getrennt in Abb. 4 dargestellt. Nur die Punktwerte der Fragen 1, 3, 5 und 9 unterscheiden sich signifikant von den Einzelpunktwerten der Patienten mit obstruktiver Tubenfunktionsstörung. Die Einzelpunktwerte der Patienten mit klaffender Tube unterscheiden sich bis auf die Fragen 5 und 9 nicht signifikant von den Einzelpunktwerten der Tinnituspatienten. In der ROC-Analyse ergibt sich für die Diagnosestellung einer klaffenden Tubenfunktionsstörung mit dem PHI-10 eine Fläche unter der Kurve von 1 (95%-Konfidenzintervall 1–1). Die Sensitivität des PHI-10 für die Diagnosestellung einer klaffenden Tube errechnet sich mit 100 %, die Spezifität mit 99 %. Der positive prädiktive Wert liegt bei 92 % und der negative prädiktive Wert bei 100 %. Die ROC-Analyse zur Unterscheidungsfähigkeit des PHI-10 zwischen der klaffenden Tube und der obstruktiven Tubenfunktionsstörung ergibt eine Fläche unter der Kurve von 0,743 (95%-Konfidenzintervall von 0,591–0,895).

Zur Verbesserung der Abgrenzungsfähigkeit des ETDQ‑7 und um falsch-positive Ergebnisse bei Tinnituspatienten möglicherweise vermeiden zu können, wurde eine Analyse des Fragebogens mit Ausschluss der Fragen 5 und 6 durchgeführt. Es ergibt sich ein neuer Fragebogen mit einem Maximalpunkwert von 35 Punkten. In einer ROC-Analyse errechnet sich bei einem Cut-off von ≥14,5 Punkten zur Diagnosestellung einer obstruktiven Tubenfunktionsstörung eine Sensitivität von 75 % und eine Spezifität von 97,5 % mit einer Fläche unter der Kurve von 0,957 (95%-Konfidenzintervall 0,921–0,993). Es errechnet sich in der ROC-Analyse zur Unterscheidung zwischen der obstruktiven Tubenfunktionsstörung und dem Tinnitus eine Fläche unter der Kurve von 0,778 (95%-Konfidenzintervall 0,653–0,903). Die Rate der falsch-positiven Ergebnisse der Tinnituspatienten sinkt in dieser Analyse von 59,09 % (*n* = 13 Ohren) auf 22,73 % (*n* = 5 Ohren). Darüber hinaus erfolgte eine Auswertung des originalen ETDQ‑7 mit Verschiebung des Cut-offs auf ≥19 Punkte zur Diagnosestellung einer obstruktiven Tubenfunktionsstörung. Die Rate der falsch-positiven Ergebnisse bei Tinnituspatienten vermindert sich in unserem Patientengut auf 27,27 % (*n* = 6 Ohren). Allerdings sinkt in dieser Analyse auch die Sensitivität auf 68,8 % bei einer Spezifität von 97,5 %. Analoge Analysen des PHI-10 unter Ausschluss einzelner Fragen und/oder Verschiebung des Cut-offs brachten keinen Verbesserungsvorteil und werden daher an dieser Stelle nicht weiter dargestellt.

## Diskussion

Die sichere Unterscheidung zwischen klaffender und obstruktiver Tubenfunktionsstörung gestaltet sich aufgrund der oft diffusen Symptomatik und des fehlenden diagnostischen Standards schwierig [10, 20, 28]. In Ermangelung guter objektiver Messverfahren wird die Diagnostik im Wesentlichen auf Anamnese und klinischen Befund gestützt. Insbesondere die Anamnese kann dabei genauso wegweisend wie irreführend sein. In der klinischen Routine konnten sich bisher weder der Tubenscore noch die Tubenmanometrie oder auch die Tympanometrie allein als ausreichend zur sicheren Diagnostik einer Tubenfunktionsstörung erweisen. Der ETDQ‑7 und der PHI-10 bieten hier eine vielversprechende Möglichkeit zur sinnvollen Unterstützung der Anamnese.

Im Jahr 2012 veröffentlichen McCoul und Mitarbeiter den Eustachian Tube Dysfunction Questionnaire (ETDQ-7) [12]. Sie präsentierten einen 7 Fragen umfassenden Fragebogen zur Diagnostik von chronisch obstruktiven Tubenfunktionsstörungen. Zugrunde gelegt wurden etablierte Fragebögen, wie z. B. der Otitis media 6‑Item Quality-of-life Survey (OM-6) [18] oder der SNOT-20 [14]. Die Antwortskala des ETDQ‑7 reicht von 1 (kein Problem) bis 7 (schwerstes Problem). Es wurden 50 erwachsene Patienten mit Tubenfunktionsstörung und 25 gesunde Erwachsene untersucht. Die Gruppenzuteilung zu Patienten mit obstruktiver Tubenfunktionsstörung erfolgte allein anhand von Trommelfellbefund und Tympanogramm. Es ergab sich ein Cronbach‑α von 0,711 (95%-Konfidenzintervall 0,570–0,818) für den gesamten ETDQ‑7 und ein Korrelationskoeffizient von r = 0,78 nach Spearman. Der ETDQ-7-Gesamtpunktwert war bei den 50 Patienten mit Tubenfunktionsstörung signifikant größer als bei den Gesunden (t = 12,2; *p* < 0,001). Der Grenzwert zur Diagnose einer Tubenfunktionsstörung wurde mit ≥14,5 Punkten bei 100 % Sensitivität und 100 % Spezifität in der ROC-Analyse angegeben [12]. Teixeira et al. untersuchten die Validität des ETDQ‑7 im Jahr 2017 unabhängig nach. Die Fläche unter der Kurve lag in dieser Analyse bei 68 %. Sie beschrieben eine höhere Korrelation des Fragebogens zu den Beschwerden als zu objektiv messbaren Parametern [27]. Wir übersetzten den ETDQ‑7 im Rahmen einer anderen Studie ins Deutsche und testeten ihn an 100 Gesunden und 43 Patienten mit chronisch obstruktiver Tubenfunktionsstörung. Der mittlere Gesamtpunktwert des ETDQ‑7 lag bei den Gesunden bei 8,67 und bei den Patienten mit chronisch obstruktiver Tubenfunktionsstörung bei 24,7. Es ergab sich eine Sensitivität des ETDQ‑7 von 90,7 % und eine Spezifität von 95 %. Der positive prädiktive Wert lag bei 88,6 % und der negative prädiktive Wert bei 96 %. Die Fläche unter der Kurve lag in unserer ROC-Analyse bei 98,8 % (*p* < 0,0001) [22]. Aus dem Jahr 2018 gibt es eine Publikation zur Übersetzung des ETDQ‑7 ins Portugiesische und eine Nachvalidierung mit 20 Patienten mit obstruktiver Tubenfunktionsstörung und einer gesunden Vergleichsgruppe. Es wurde eine Sensitivität von 95 % und eine Spezifität von 97 % erreicht [2]. Eine ähnliche Untersuchung mit der chinesischen Übersetzung ergab in der ROC-Analyse eine AUC von 99,8 % mit Sensitivität 100 % und Spezifität 99,9 % [9].

Im Jahr 2017 wurde ein Scoringsystem bei klaffender Tube von Kobayashi und Mitarbeitern veröffentlicht. Der PHI-10 enthält 10 Fragen bezogen auf die Symptome der letzten 4 Wochen, und die Einteilung des Schweregrads der klaffenden Tube richtet sich nach dem erreichten Gesamtpunktwert. Jede einzelne Frage kann mit „ja“, entsprechend 4 Punkten, „manchmal“, entsprechend 2 Punkten, und „nein“, entsprechend 0 Punkten, beantwortet werden. Ein Gesamtpunktwert von 0–8 wird als keine Beeinträchtigung, ein Gesamtpunktwert von 10–16 als milde Beeinträchtigung, ein Gesamtpunktwert von 18–24 als mäßige Beeinträchtigung und ein Gesamtpunktwert von 26–40 als schwere Beeinträchtigung gewertet. Es wurden 31 Patienten mit klaffender Tube und Therapie mit dem Kobayashi Plug, 29 Patienten mit konservativer Therapie einer klaffenden Tube und 29 Patienten mit Schallempfindungsschwerhörigkeit untersucht. Die Diagnose einer klaffenden Tube wurde anhand der Diagnosekriterien der japanischen Gesellschaft für Otologie gestellt. Zur Bestimmung der Reliabilität wurde die interne Konsistenz mit einem Cronbach‑α von 0,887 für alle Fragen berechnet [4].

Beide Fragebögen sind kurz und einfach in der Anwendung. Sie können schon vor dem ersten Arzt-Patienten-Kontakt im Warteraum ausgefüllt werden. So kann das Anamnesegespräch vorbereitet werden. Die Ergebnisse unserer Untersuchung zeigen, dass sowohl der ETDQ‑7 als auch der PHI-10 in der Lage sind, gesunde Probanden und Patienten mit Tubenfunktionsstörungen sicher zu unterscheiden. Eine genaue Differenzierung zwischen klaffender und obstruktiver Tubenfunktionsstörung gelingt hingegen nicht zuverlässig. Zudem traten falsch-positive Ergebnisse bei Tinnituspatienten auf. Diese sollten also grundsätzlich nicht oder nur mit entsprechendem Wissen mit dem ETDQ‑7 und PHI-10 untersucht werden. Die Stärke der Fragebögen sehen wir deshalb in der Verlaufs- und Therapieerfolgskontrolle nach Behandlungsversuchen von Tubenfunktionsstörungen. Untersuchungen an größeren Kollektiven müssen zeigen, ob ein Ausschluss der Fragen 5 und 6 des ETDQ‑7 oder die Verschiebung des Cut-offs tatsächlich die Unterscheidungsfähigkeit des ETDQ‑7 verbessern können und wie dadurch Sensitivität und Spezifität beeinflusst werden.

Es gibt bisher nur wenige vergleichbare Untersuchungen des ETDQ‑7 mit Fokus auf das Antwortverhalten von Patienten mit klaffender Tubenfunktionsstörung. Im Jahr 2015 untersuchten Van Roeyen und Mitarbeiter 39 Patienten mit obstruktiver Tubenfunktionsstörung, 8 Patienten mit klaffender Tube und 22 Gesunde als Kontrollgruppe mit dem ETDQ‑7. Der mittlere Gesamt-ETDQ‑7 lag in der Kontrollgruppe mit 9,91 Punkten im Normbereich. Patienten mit obstruktiver Tubenfunktionsstörung erreichten im Mittel 25,77 Punkte und Patienten mit klaffender Tube 27 Gesamtpunkte. Die Fläche unter der Kurve lag bei 95 % für obstruktive Tubenfunktionsstörungen und sogar bei 96 % bei klaffender Tube. Eine Unterscheidung zwischen klaffender und obstruktiver Tubenfunktionsstörung gelang also nicht [17]. Ikeda und Mitarbeiter untersuchten im Jahr 2018 Patienten mit klaffender Tube und eine gesunde Vergleichsgruppe mit dem ETDQ‑7. Auch hier wurde eine hohe Rate falsch-positiver Ergebnisse bei Patienten mit klaffender Tube festgestellt. Der mittlere ETDQ‑7 lag in der gesunden Kontrollgruppe bei 7,6 Punkten und bei Patienten mit klaffender Tube bei 22,5 Punkten und damit im pathologischen Bereich [3]. Eine Studie aus dem Jahre 2018 von Smith et al. bestätigte dies ebenfalls [26].

Es gibt mit dem 10-item Cambridge Eustachian Tube Dysfunction Assessment (CETDA) inzwischen einen weiteren Fragebogen zur Tubenfunktion, der insbesondere als Instrument zur Therapieüberwachung konzipiert wurde [26]. Auch dieser kann nicht sicher zwischen klaffender und obstruktiver Tubenfunktionsstörung unterscheiden [25] und wurde in dieser Arbeit nicht berücksichtigt.

Zum PHI-10 liegen bisher kaum Publikationen vor. In einer Arbeit von Kawamura et al. aus dem Jahr 2019 wurden 74 Ohren mit klaffender Tube mit dem PHI-10 untersucht. Er wurde lediglich zur Schweregradeinteilung genutzt und nicht als eigenständiges diagnostisches Instrument. Dabei gaben 12,2 % keine relevanten Beschwerden im PHI-10 an und 58,1 % eine höchstgradige Beeinträchtigung [6].

## Fazit für die Praxis

Wir empfehlen beide Fragebögen, den ETDQ‑7 und den PHI-10, zur Unterstützung der gezielten Anamnese bei Verdacht auf Tubenfunktionsstörungen und als zusätzliches Instrument zur Verlaufskontrolle nach Therapie. Der Anwender muss allerdings berücksichtigen, dass nicht sicher zwischen klaffender und obstruktiver Tubenfunktionsstörung unterschieden werden kann. Für Patienten, die zusätzlich unter einem Tinnitus leiden, können beide Fragebögen nicht verlässlich eingesetzt werden.
