# Erratum zu: Untersuchung standardisierter Anamnesefragebögen zur Diagnostik und Differenzierung von obstruktiven und klaffenden Tubenfunktionsstörungen

**DOI:** 10.1007/s00106-021-01083-4

**Published:** 2021-07-13

**Authors:** J. Lönnecker, N. M. Weiss, A. Heinrichs, R. Mlynski, S. Rettschlag

**Affiliations:** Klinik für Hals-Nasen-Ohrenheilkunde, Kopf- und Halschirurgie „Otto Körner“, Universitätsmedizin Rostock, Doberaner Straße 137–139, 18057 Rostock, Deutschland

**Erratum zu:**

**HNO 2020**

10.1007/s00106-020-00931-z

Der Artikel „Untersuchung standardisierter Anamnesefragebögen zur Diagnostik und Differenzierung von obstruktiven und klaffenden Tubenfunktionsstörungen“ von J.J. Lönnecker, N.M. Weiss, A. Heinrichs, R. Mlynski und S. Rettschlag wurde ursprünglich Online First ohne „Open Access“ auf der Internetplattform des Verlags publiziert. Nach der Veröffentlichung in Band 69 Heft 3 pp. 198–205 hatten sich die Autoren für eine „Open Access“-Veröffentlichung entschieden. Das Urheberrecht des Artikels wurde deshalb in © Der/die Autoren 2020 geändert.

Dieser Artikel wird nun unter der Creative Commons Namensnennung 4.0 International Lizenz veröffentlicht, welche die Nutzung, Vervielfältigung, Bearbeitung, Verbreitung und Wiedergabe in jeglichem Medium und Format erlaubt, sofern Sie den/die ursprünglichen Autor(en) und die Quelle ordnungsgemäß nennen, einen Link zur Creative Commons Lizenz beifügen und angeben, ob Änderungen vorgenommen wurden. Die in diesem Artikel enthaltenen Bilder und sonstiges Drittmaterial unterliegen ebenfalls der genannten Creative Commons Lizenz, sofern sich aus der Abbildungslegende nichts anderes ergibt. Sofern das betreffende Material nicht unter der genannten Creative Commons Lizenz steht und die betreffende Handlung nicht nach gesetzlichen Vorschriften erlaubt ist, ist für die oben aufgeführten Weiterverwendungen des Materials die Einwilligung des jeweiligen Rechteinhabers einzuholen. Weitere Details zur Lizenz entnehmen Sie bitte der Lizenzinformation auf http://creativecommons.org/licenses/by/4.0/deed.de.

